# Muscle-strengthening activities and cancer incidence and mortality: a systematic review and meta-analysis of observational studies

**DOI:** 10.1186/s12966-021-01142-7

**Published:** 2021-05-29

**Authors:** Wilson Nascimento, Gerson Ferrari, Camila Bertini Martins, Juan Pablo Rey-Lopez, Mikel Izquierdo, Dong Hoon Lee, Edward L. Giovannucci, Leandro F. M. Rezende

**Affiliations:** 1 Universidade Federal de São Paulo, Escola Paulista de Medicina, Departamento de Medicina Preventiva, São Paulo, SP Brazil; 2 Universidad de Santiago de Chile (USACH), Escuela de Ciencias de la Actividad Física, el Deporte y la Salud, Santiago, Chile; 3 Faculty of Health Sciences, International University of Valencia (VIU), Valencia, Spain; 4 Faculty of Sport, Catholic University San Antonio of Murcia, Murcia, Spain; 5 Navarrabiomed, Navarra Hospital Complex (NHC), Universidad Pública de Navarra (UPNA), IdiSNA, Pamplona, Spain; 6 Department of Nutrition, Harvard T.H. Chan School of Public Health, Boston, MA USA; 7 Department of Epidemiology, Harvard T.H. Chan School of Public Health, Boston, MA USA; 8 Channing Division of Network Medicine, Department of Medicine, Brigham and Women’s Hospital and Harvard Medical School, Boston, MA USA

**Keywords:** Physical activity, Muscle-strengthening activities, Neoplasia, Meta-analysis

## Abstract

**Background:**

Physical activity has been associated with reduced risk of seven types of cancer. It remains unclear, however, whether muscle-strengthening activities also reduce cancer incidence and mortality.

**Methods:**

PubMed, Embase, Web of Science and Scopus were searched from inception to March 2020. Summary hazard ratio (HR) and 95% confidence intervals (CI) were estimated using random-effects models.

**Results:**

Twelve studies (11 cohorts; 1 case-control), 6 to 25 years of follow-up, including 1,297,620 participants, 32,196 cases and 31,939 deaths, met inclusion criteria. Muscle-strengthening activities were associated with a 26% lower incidence of kidney cancer (HR for high vs low levels of muscle-strengthening activities: 0.74; 95% CI 0.56 to 0.98; *I*^2^ 0%; 2 studies), but not with incidence of other 12 types of cancer. Muscle-strengthening activities were associated with lower total cancer mortality: HRs for high vs low levels of muscle-strengthening activities was 0.87 (95% CI 0.73 to 1.02; *I*^2^ 58%; 6 studies); and HR for ≥2 times/week vs < 2 times/week of muscle-strengthening activities was 0.81 (95% CI 0.74 to 0.87; *I*^2^ 0%; 4 studies). Regarding the weekly duration of muscle-strengthening activities, HR for total cancer mortality were 0.91 (95% CI 0.82 to 1.01; *I*^2^ 0%; 2 studies) for 1–59 min/week and 0.98 (95% CI 0.89 to 1.07; *I*^2^ 0%) for ≥60 min/week vs none. Combined muscle-strengthening and aerobic activities (vs none) were associated with a 28% lower total cancer mortality (HR 0.72; 95% CI 0.53 to 0.98; *I*^2^ 85%; 3 studies).

**Conclusions:**

Muscle-strengthening activities were associated with reduced incidence of kidney cancer and total cancer mortality. Combined muscle-strengthening and aerobic activities may provide a greater reduction in total cancer mortality.

**Supplementary Information:**

The online version contains supplementary material available at 10.1186/s12966-021-01142-7.

## Introduction

Physical activity has received much attention in recent years for its potential to prevent some types of cancer [1–3]. In 2018, the *World Cancer Research Fund/American Institute of Cancer Research* concluded that convincing evidence exist to support that aerobic, moderate to vigorous physical activity (MVPA) is associated with a reduced risk of colon cancer, and there is probable evidence for a reduction in the risk of breast cancer and endometrial cancer [3]. More recently, the *American College of Sports Medicine* [1] *and the United States Department of Health and Human Services* [4] have concluded strong evidence for the relationship between aerobic MVPA and reduced risk of seven types of cancer: breast, colon, endometrial, esophagus, kidney, bladder and stomach. Nevertheless, the potential effect of different types of physical activity for cancer prevention remains unclear [5].

Muscle-strengthening activities are activities that maintain or increase the physical fitness component of muscle strength, as well as body composition, balance and muscular endurance [6]. The 2020 *World Health Organization* (WHO) physical activity recommendations for health included muscle-strengthening activities involving major muscle groups during at least twice weekly [7], due to their association with a probable reduced risk of cardiovascular mortality [8, 9], type 2 diabetes [10] and all-cause mortality [9]. Yet, the relationship between muscle-strengthening activities and cancer incidence and mortality is uncertain. Recent large prospective cohort studies have reported an association between muscle-strengthening activities with lower total cancer mortality [11], and incidence of colon [12] and kidney cancers [13], but not for other types of cancer [12, 13].

Given these favorable results of muscle-strengthening activities in cancer prevention, we conducted a systematic review to assess the epidemiologic evidence on 1) the association of muscle-strengthening activities with cancer incidence and mortality by types of cancer; 2) the parameters (type, duration, frequency and intensity) of muscle-strengthening activities needed to reduce cancer incidence and mortality; 3) the joint association of muscle-strengthening activities and aerobic MVPA with cancer incidence and mortality; and 4) the association of muscle-strengthening activities with cancer incidence and mortality according to potential effect modifiers.

## Methods

This systematic review was registered under PROSPERO (CRD42020153846) and the reporting followed the PRISMA 2020 checklist recommendations [14].

### Literature search and study selection

We conducted a broad search, without publication date or language filters, in MEDLINE (via Pubmed), Embase, Web of Science and Scopus databases in March 2020, using search terms related to exposure (“strength training”) and outcome (“cancer”). Details on the search strategy can be found in the supplementary material.

Our systematic review aimed to investigate whether muscle-strengthening activities (exposure) can reduce the risk of cancer incidence and mortality (outcome) in participants without cancer at baseline (population). Specifically, our research question addresses primary prevention: Do muscle-strengthening activities prevent cancer incidence and mortality in healthy populations? Observational studies (case-control and cohort) that evaluated the association between muscle-strengthening activities, alone or combined with aerobic MVPA, and cancer incidence or mortality, were eligible for inclusion. The studies should contain information on muscle-strengthening activities (e.g., weight training, weightlifting) in healthy adults (≥18 years) free of cancer at baseline (for cohort studies). Theoretically, randomized clinical trials were eligible for our analysis, but yielded no results, probably due to logistical and economic issues. Congress abstracts, narrative reviews and cross-sectional studies were ineligible. We excluded cohort studies that included participants with cancer at baseline as they address an entirely different research question – secondary/tertiary prevention: Do muscle-strengthening activities improve cancer survival (reduce total and cancer-specific mortality) in populations with a diagnosis of cancer? Selection of articles first involved reading and evaluating titles and abstracts considering the scope of the systematic review. Then, the full texts were read for the final selection. In both stages, the articles were selected by two researchers (WN and GF) and compared; in cases of disagreement, a third researcher (LFMR) was asked to arbitrate.

### Data extraction

Data were extracted on study characteristics (year of publication and study design), participants (sample size and sociodemographic characteristics), exposure (type, frequency, duration and intensity of the muscle-strengthening activities), muscles used, follow-up time (for cohort studies), outcomes (cancer incidence and mortality; types of cancer) and results (e.g., number of cases/deaths, multivariable-adjusted hazard ratios [HR] and 95% confidence intervals [CI]). When the information was unavailable in the study, we contacted the authors to complement data extraction.

### Risk of bias assessment

Two researchers (DHL and JPRL) independently assessed the risk of bias in each study using the ROBINS-I tool [15], which included the following bias domains: (1) confounding; (2) bias in the sample selection of the study; (3) bias in the classification of the intervention/exposure; (4) deviations from the intended exposure; (5) bias in the classification of the outcome; (6) bias due to missing data; (7) selection of the reported results. Any disagreement in the assessment was discussed with and resolved by a third researcher (LFMR).

### Statistical analysis

Fixed and random effect meta-analyses were performed to estimate summary measures (HR and 95% CI) for the association between high vs low levels of muscle-strengthening activities and cancer incidence and mortality by types of cancer. “High” was defined as the group with the highest volume (or frequency) of muscle-strengthening activities, whereas “low” was the group that reported none or lower volume (or frequency) of muscle-strengthening activities. Subgroup analyses were performed with studies that used similar criteria for categorizing exposure (e.g., ≥2 times/week vs < 2 times/week; ≥60 min/week, 1–59 min/week vs none). Whenever possible, a meta-analysis was conducted to estimate the summary HR and 95% CI for the joint association of muscle-strengthening activities (e.g., ≥2 times/week vs < 2 times/week; Any vs None) and aerobic MVPA (e.g., ≥150 vs < 150 min/week) with cancer incidence and mortality. Heterogeneity between study results was quantified by *I*^2^ and Cochran’s Q statistics [16]. The systematic review protocol included assessing small study effects (publication bias) [17] and sources of heterogeneity between studies; but due to the limited number of eligible studies included in the review, these tests were not performed. All statistical analyses were performed using RStudio, version 1.2.5042.

## Results

**Fig. 1:**
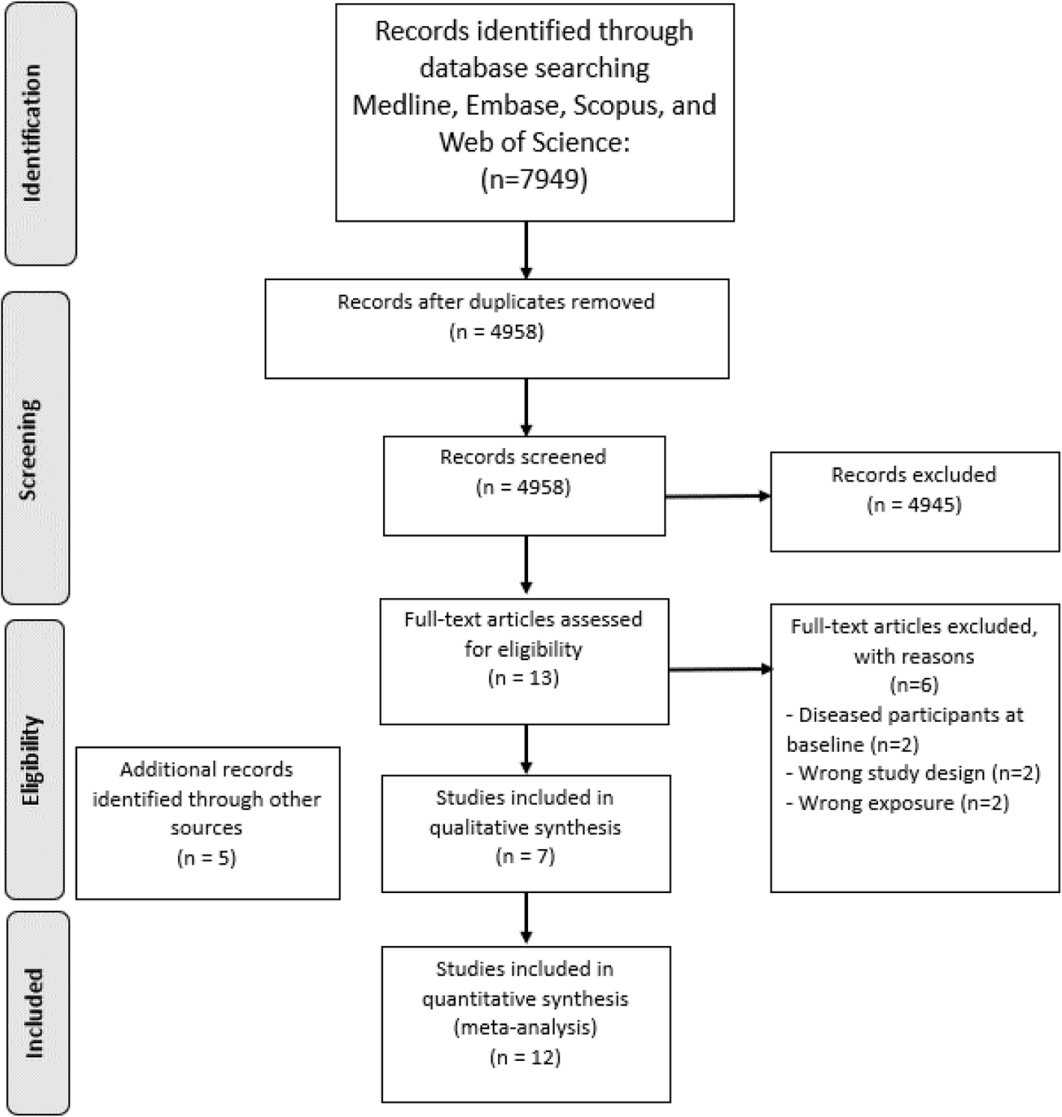
Preferred Reporting Items for Systematic Reviews and Meta-analysis (PRISMA) flow diagram for search strategy

Search strategy was performed in four databases (MEDLINE [via Pubmed], Embase, Web of Science and Scopus) and duplicates were removed, obtaining a total of 4958 articles. Of these, 7 met the eligibility criteria for the systematic review. An additional manual search from the lists of recent reviews and included studies, as well as the continuous update from Pubmed alerts yielded 5 eligible studies. Finally, the systematic review included 12 studies [8, 11–13, 18–25] (Fig. 1).

Summarized characteristics of all studies selected in the systematic review are shown in Table S1. Of the 12 articles, eleven were cohort studies [8, 11–13, 18–21, 23–25] and one case-control study [22]; ten studies [8, 12, 13, 18–21, 23–25] were conducted in the US, one in Australia [22] and one in the United Kingdom (England and Scotland) [11]. All studies measured muscle-strengthening activities via questionnaires. Regarding sample classification, several studies classified participants according to the weekly frequency of muscle-strengthening activities [11, 18, 20, 21, 24, 25], others used weekly duration [8, 12, 13, 19] and equivalent of tasks [22, 23]. A detailed definition of muscle-strengthening activities by type of measure, type of activity and analytical categories across the 12 studies is available in Table S1. Regarding the outcomes, eight articles assessed total cancer mortality [8, 11, 18–21, 24, 25] and four assessed cancer incidence according to primary tumor location, as follows: colon [12, 13, 22], prostate [12, 13], lung [12, 13], kidney [12, 13], lymphoma [12, 13], pancreas [12, 13], multiple myeloma [13], bladder [12, 13], esophagus [12, 13], rectum [12, 22], melanoma [12], leukemia [13] cancers of the digestive system [23].

Regarding the risk of bias, for the confounding domain, 3 studies were judged as low risk of bias [8, 13, 23], eight with serious risk of bias [11, 12, 18–21, 24, 25] and one as critical risk of bias [22] (Table 1). For three domains (selection of participants [8, 11–13, 19, 21, 23, 24], classification of the exposures [8, 11–13, 18–21, 24, 25] and measurement of the outcomes [8, 11–13, 18–21, 23–25]), most of the studies were judged as low risk of bias. For the domain deviations from intended exposures, most of the studies were evaluated as moderate risk of bias [8, 11–13, 18–21, 23–25]. Regarding the missing data domain, four studies were judged as no information [11, 20, 24, 25], three with moderate risk of bias [12, 19, 22], and five with low risk of bias [8, 13, 18, 21, 23]. For the selection of the reported results domain, most of the studies were scored as moderate risk of bias [8, 11–13, 18–21, 23–25] and one as serious risk [22].

**Table 1:** Risk of bias judgement by domains of bias using the ROBINS-I tool

	Confounding^a^	Selection of participants	Classification of exposure	Deviations from intended exposures	Missing data	Measurement of outcomes	Selection of the reported result
Darkel, 2016 [25]	Serious	Moderate	Low	Moderate	Ni	Low	Moderate
Boyle, 2012 [22]	Critical	Serious	Serious	Serious	Moderate	Moderate	Serious
Kamada, 2017 [8]	Low	Low	Low	Moderate	Low	Low	Moderate
Keum, 2016 [23]	Low	Low	Moderate	Moderate	Low	Low	Moderate
Kraschnewski, 2016 [18]	Serious	Moderate	Low	Moderate	Low	Low	Moderate
Loprinzi, 2017 [20]	Serious	Critical	Low	Moderate	Ni	Low	Moderate
Mazzilli, 2019 [12]	Serious	Low	Low	Moderate	Moderate	Low	Moderate
Patel, 2020 [19]	Serious	Low	Low	Moderate	Moderate	Low	Moderate
Rezende, 2020 [13]	Low	Low	Low	Moderate	Low	Low	Moderate
Siahpush, 2018 [24]	Serious	Low	Low	Moderate	Ni	Low	Moderate
Stamatakis, 2018 [11]	Serious	Low	Low	Moderate	Ni	Low	Moderate
Zhao, 2020 [21]	Serious	Low	Low	Moderate	Low	Low	Moderate

^a^ A list of confounders considered in the assessment of risk of confounding: age, sex, smoking, adiposity, alcohol consumption, dietary factors, individual-level socioeconomic factors, and other aerobic physical activities

### Associations of muscle-strengthening activities with cancer incidence and mortality

**Fig. 2:**
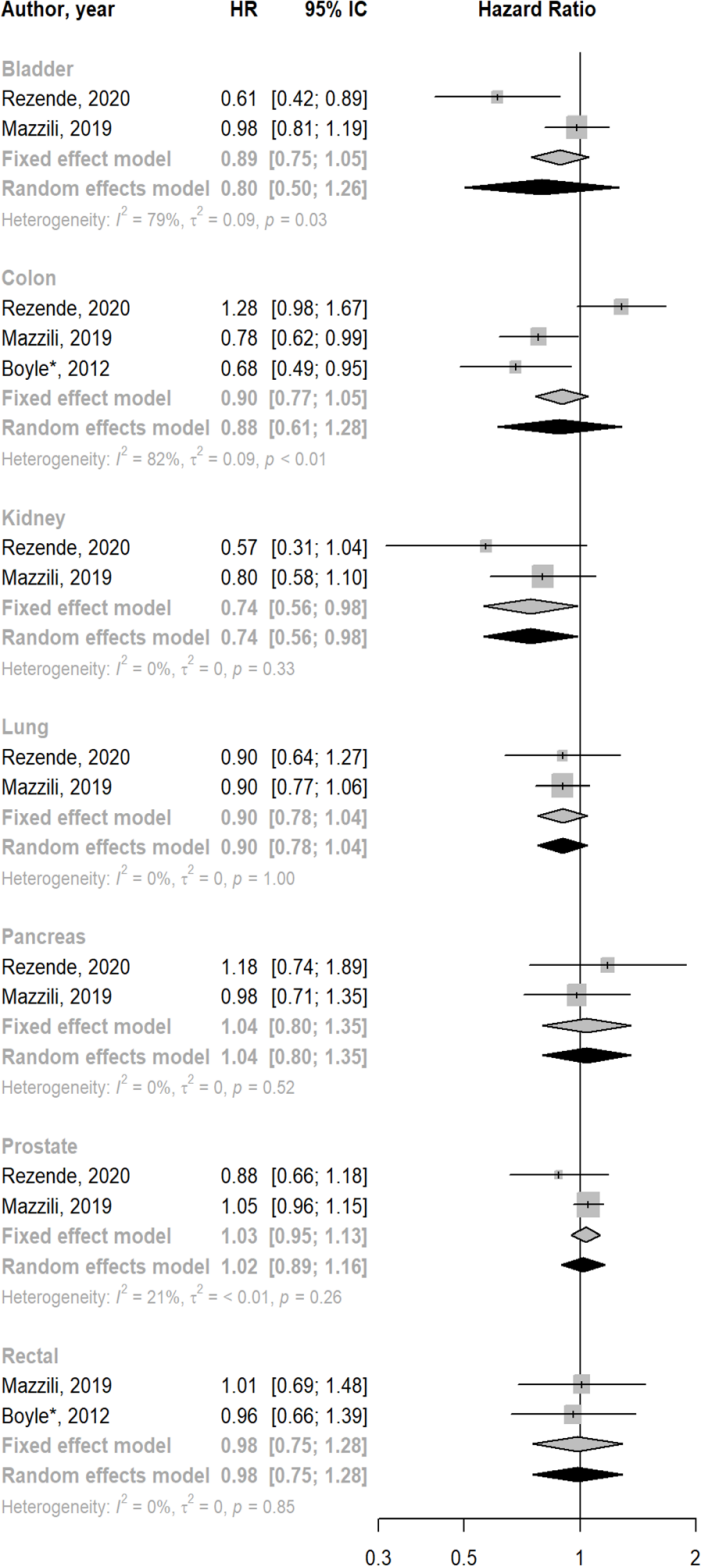
Meta-analysis for the association between high vs low muscle-strengthening activities and cancer incidence by type of cancer. *Footnote: Case-control study

Three studies [12, 13, 22] examined the association between muscle-strengthening activities and incidence of colon cancer, totaling 250,775 participants and 2967 cases. The summary HR for the association between high vs low levels of muscle-strengthening activities and incidence of colon cancer was 0.88 (95% CI: 0.61 to 1.28; I^2^ 82%) (Fig. 2).

Rezende et al. [13] and Mazzilli et al. [12] evaluated the association between muscle-strengthening activities and the incidence of ten types of cancer. The summary HR for high vs low levels of muscle-strengthening activities and cancer incidence was 0.80 (95% CI: 0.50 to 1.26; *I*^2^ 79%) for bladder cancer, 0.74 (95% CI: 0.56 to 0.98; *I*^2^ 0%) for kidney cancer, 0.90 (95% CI: 0.78 to 1.04; *I*^2^ 0%) for lung cancer, 1.04 (95% CI: 0.80 to 1.35; *I*^2^ 0%) for pancreatic cancer, and 1.02 (95% CI: 0.89 to 1.16; *I*^2^ 21%) for prostate cancer (Fig. 2).

Mazzilli et al. [12] and Boyle et al. [22] evaluated the association between muscle-strengthening activities and the incidence of rectal cancer. The summary HR for the association between high vs low levels of muscle-strengthening activities and incidence of rectal cancer was 0.98 (95% CI: 0.75 to 1.28; *I*^2^ 0%) (Fig. 2).

Individual studies reported null associations between muscle-strengthening activities and the incidence of lymphoma [13], non-Hodgkin’s lymphoma [12, 13], leukemia [13], multiple myeloma [13], melanoma [12] and esophageal cancer [13].

**Fig. 3:**
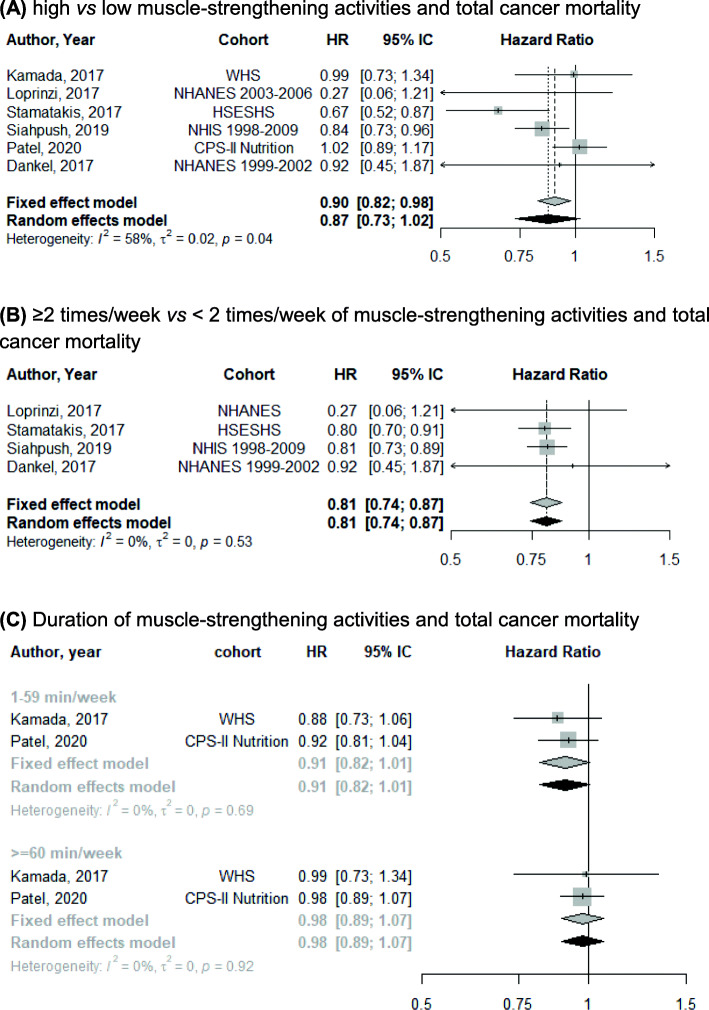
Meta-analysis for the association between **A** high vs low; **B** weekly frequency; and **C** duration of muscle-strengthening activities and total cancer mortality

Six studies [8, 11, 19, 20, 24, 25] were included in the meta-analysis for the association between muscle-strengthening activities and total cancer mortality, totaling 493,348 participants and 15,372 deaths. The summary HR for the association between high vs low levels of muscle-strengthening activities and total cancer mortality was 0.87 (95% CI 0.73 to 1.02; *I*^2^ 58%) (Fig. 3A). Four studies [11, 20, 24, 25] used the criterion ≥2 times/week vs < 2 times/week of muscle-strengthening activities, with a summary HR for total cancer mortality of 0.81 (95% CI 0.74 to 0.87; I^2^ 0%) (Fig. 3B). Two studies [8, 19] assessed the weekly duration of muscle-strengthening activities. Compared with participants who performed no muscle-strengthening activities, the summary HR for total cancer mortality was 0.91 (95% CI 0.82 to 1.01; *I* ^2^ 0%) for 1–59 min/week and 0.98 (95% CI 0.89 to 1.07; *I* ^2^ 0%) for ≥60 min/week (Fig. 3C).

### Joint association of muscle-strengthening and aerobic activities with cancer incidence and mortality

**Fig. 4:**
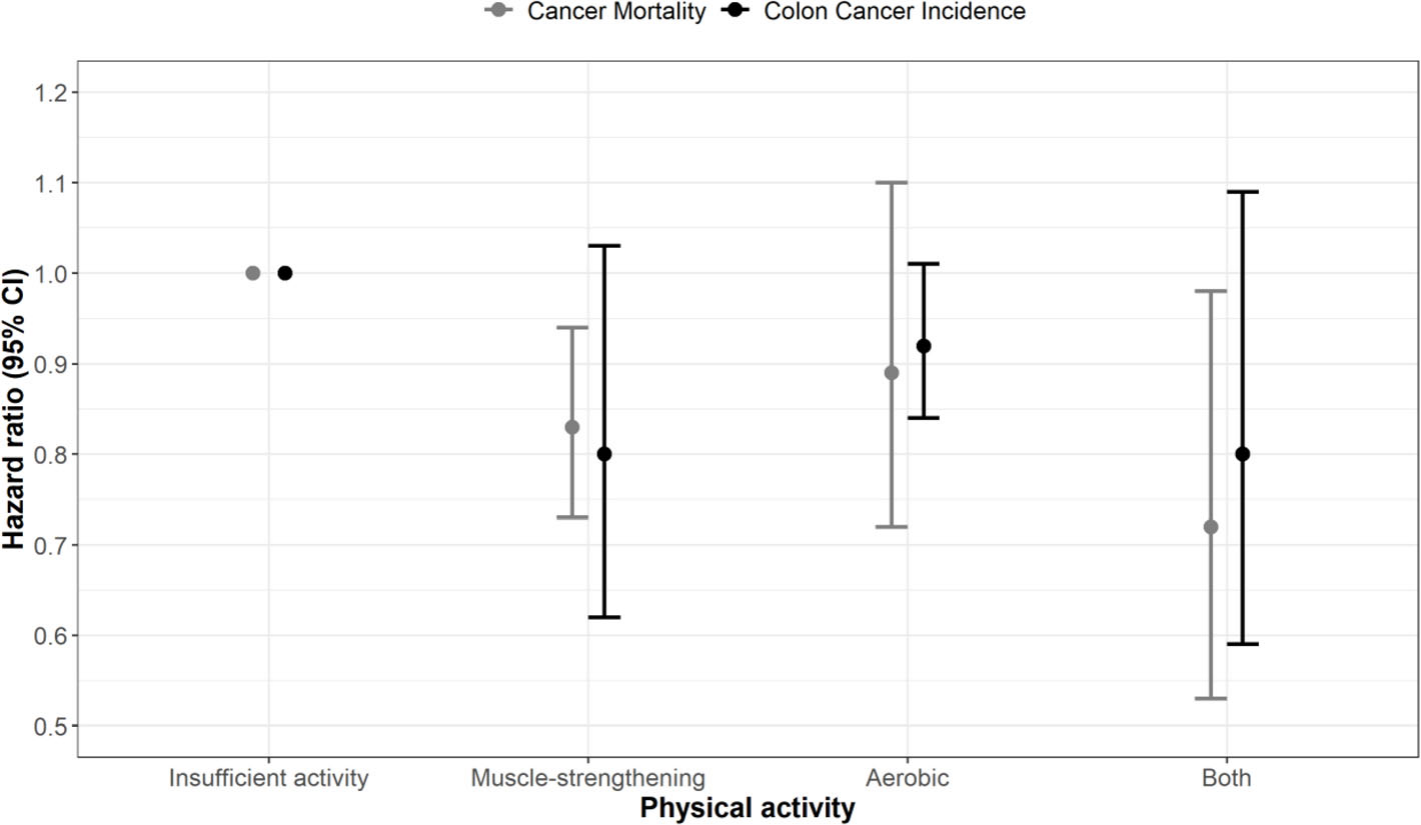
Meta-analysis for the joint association of muscle-strengthening and aerobic activities with colon cancer incidence and total cancer mortality. Footnote: Three studies performed joint association of muscle-strengthening activities and aerobic MVPA with colon cancer incidence and total cancer mortality. The muscle-strengthening and aerobic activity groups were defined as follow: Total Cancer Mortality: *Kamada* et al: Insufficient activity (Reference): < 150 min/week of aerobic MVPA and no strength training; Aerobic activities only: Aerobic MVPA ≥150 min/week and no strength training; Muscle-strengthening activities only: < 150 min/week of aerobic MVPA and any strength training; Both: Aerobic MVPA ≥150 min/week and any strength training; *Stamatakis* et al: Insufficient activity (Reference): < 150 min/week of aerobic MVPA and < 2 times/week of strength training; Aerobic activities only: Aerobic MVPA ≥150 min/week and < 2 times/week of strength training; Muscle-strengthening activities only: < 150 min/week of aerobic MVPA and ≥ 2 times/week of strength training; Both: Aerobic MVPA ≥150 min/week and ≥ 2 times/week of strength training.; *Zhao* et al.: Insufficient activity (Reference): < 150 min of light to moderate intensity activity each week, or < 75 min of vigorous intensity activity, or less than an equivalent combination and < 2 times/week of muscle-strengthening activities; Aerobic activities only: ≥150 min of light to moderate intensity activity each week, or ≥ 75 min of vigorous intensity activity, or greater than or equal to an equivalent combination and < 2 times/week of muscle-strengthening activities; Muscle-strengthening activities only: < 150 min of light to moderate intensity activity each week, or < 75 min of vigorous intensity activity, or less than an equivalent combination and ≥ 2 times/week of muscle-strengthening activities; Both: ≥150 min of light to moderate intensity activity each week, or ≥ 75 min of vigorous intensity activity, or greater than or equal to an equivalent combination and ≥ 2 times/week of muscle-strengthening activities. Colon cancer incidence: *Rezende* et al: Insufficient activity (Reference): < 16 MET-h/week of aerobic MVPA and no resistance training; Aerobic activities only: ≥16 MET-h/week of aerobic MVPA and no resistance training; Muscle-strengthening activities only: < 16 MET-h/week of aerobic MVPA and any resistance training; Both: ≥16 MET-h/week of aerobic MVPA and any resistance training. *Mazzilli* et al: Insufficient activity (Reference): < 7.5 MET-h/week of aerobic MVPA and no weight lifting; Aerobic activities only: ≥7.5 MET-h/week of aerobic MVPA and no resistance tr no weight lifting; Muscle-strengthening activities only: < 7.5-h/week of aerobic MVPA and any weight lifting; Both: ≥7.5 MET-h/week of aerobic MVPA and any weight lifting

Two studies [12, 13] assessed the joint association of muscle-strengthening activities and aerobic MVPA with colon cancer incidence, totaling 248,909 participants and 2415 cases. Compared with participants that performed neither muscle-strengthening activities nor aerobic MVPA, the summary HR for colon cancer incidence was 0.80 (95% CI 0.62 to 1.03; *I*^2^ 0%) for muscle-strengthening activities only, 0.92 (95% CI 0.84 to 1.01; *I*^2^ 0%) for aerobic MVPA only, and 0.80 (95% CI 0.59 to 1.09; *I*^2^ 83%) for muscle-strengthening activities plus aerobic MVPA (Fig. 4; Figure S1).

Individual studies found that combined muscle-strengthening activities and aerobic MVPA (vs none) was associated with a lower incidence of kidney [13] and bladder [13] cancers, but not with cancers of the digestive system [23].

Three studies [8, 11, 21] examined the joint association of muscle-strengthening activities and aerobic MVPA with total cancer mortality, with a total of 585,930 participants and 17,212 cancer deaths. Compared with participants who performed neither muscle-strengthening activities nor aerobic MVPA, the summary HR for total cancer mortality was 0.83 (95% CI 0.73 to 0.94; *I*^2^ 21%) for muscle-strengthening activities only, 0.89 (95% CI 0.72 to 1.10; *I*^2^ 90%) for aerobic MVPA only, and 0.72 (95% CI 0.53 to 0.98; I^2^ 85%) for muscle-strengthening activities plus aerobic MVPA (Fig. 4; Figure S2).

## Discussion

Our systematic review suggested that muscle-strengthening activities were associated with lower incidence of kidney cancer. Pooled results from two large prospective cohort studies [12, 13] suggested that muscle-strengthening activities were associated with a 26% lower incidence of kidney cancer when comparing high vs low levels of muscle-strengthening activities. Results for the other 12 types of cancer included in our systematic review were inconclusive due to limited number of studies. For instance, among the same aforementioned cohort studies [12, 13] that evaluated the association between muscle-strengthening activities and incidence of colon cancer, Mazzilli et al. [12] found an inverse association with muscle-strengthening activities, whereas Rezende et al. [13], found a positive association. Of note, this positive association was restricted to the subgroup of ever smokers and participants with overweight/obesity. In addition, joint analysis between muscle-strengthening activities and aerobic MVPA suggested a null association with colon cancer [13].

We found that muscle-strengthening activities were associated with a 13% lower total cancer mortality and that joint muscle-strengthening activities and aerobic MVPA with a 28% lower total cancer mortality. A previous meta-analysis found that muscle-strengthening activities were also associated with lower all-cause and cardiovascular disease mortality [9]. The amount of muscle-strengthening activities required to achieve optimum cancer prevention remains, however, unclear. Four studies [11, 20, 24, 25] that categorized the frequency of muscle-strengthening activities as ≥2 times/week (vs < 2 times/week) found a 19% lower total cancer mortality. Two studies [8, 19] observed that, as compared with participants who performed no muscle-strengthening activities, those practicing 1–59 min/week had a 9% non-statistically significant lower cancer mortality. Nevertheless, there was a null association with total cancer mortality in participants doing ≥60 min/week of muscle-strengthening activities vs none, possibly due to limited number of events when considering this contrast.

Deciphering the causal contribution of a specific type of physical activity on cancer prevention is challenging. Low within-population variability and measurement error for physical activity are important concerns [26, 27]. Future prospective cohort studies with objective measures of physical activity may reduce misclassification and, consequently, reduce current uncertain evidence. That being said, the current technology of accelerometers cannot accurately capture the muscle-strengthening activities for scientific research purposes. Thus, a combination of questionnaires, diaries and heart rate monitoring will likely be more useful to reduce measurement error. In the meantime, results from large pooled consortia using questionnaires [28] may boost the current scientific evidence, particularly for less common cancers.

The biological mechanisms whereby muscle-strengthening activities prevent cancer are not yet fully explained, but a few hypotheses have been proposed. Some of these hypotheses may not be limited to muscle-strengthening activities, and would encompass other types of exercise and aerobic activities. The first relates to the direct benefits of muscle-strengthening activities on body composition, as a high level of body fat is directly related to the incidence of cancer. These same biological mechanisms have also been postulated for the association between aerobic MVPA and cancer incidence [29]. Excess adiposity causes insulin resistance, which in turn leads to an elevated levels of bioactive insulin-like growth factor-1 (IGF-1); both insulin and IGF-1 can increase proliferation and reduce apoptosis in sensitive cells [30]. Obesity is often accompanied by unfavorable changes to the intestinal microbiota, increasing the production of pro-inflammatory factors and some hormones, such estrogen, an important factor for breast cancer [30, 31]. Of note, most studies included in our systematic review adjusted for BMI in the multivariable models, which may have attenuated the magnitude of the associations.

A second hypothesis is that muscle-strengthening activities enhance muscle mass, which in turn enhances glucose control [10, 32]. High intensity strength training may also lead to a specific increase in free radicals, which could trigger adaptations in the body by generating more antioxidants, and may have an impact on epigenetic factors, such as DNA methylation [32, 33]. Additionally, enhanced muscle mass improves immunity, with some studies showing an improvement in natural killer cell activity, which could bolster anti-tumor reaction [32, 33]. Finally, physical activity improves microcirculation thus reducing hypoxic environments, which are important for tumor development: in their presence, tumor cells increase the expression of vascular endothelial growth factor, consequently, increasing the vascularization of the neoplastic masses [32, 34]. Of note, because most of these biological pathways are related to overall physical activity, future mechanistic studies may uncover causal links between muscle-strengthening activities and cancer incidence and mortality.

Our systematic review has some limitations that warrant consideration. First, results from meta-analyses are prone to measurement errors inherent in the included studies. Measurement error in muscle-strengthening activities could have affected the magnitude of the associations. In this case, as most of the studies included in our systematic review were prospective cohort studies, we expect that measurement error of muscle-strengthening activities being non-differential in regard to the outcome ascertained; thus, non-differential measurement error of the exposure may have underestimated the magnitude of the associations of high versus low levels of muscle-strengthening activities with cancer incidence and mortality. All studies included in our systematic review were based on participants’ self-report, so the actual intensity or duration of the muscle-strengthening activities performed cannot be established. Second, the studies reviewed used different questions to assess participation in muscle-strengthening activities and different analytical categories. This may have increased the statistical heterogeneity between studies, e.g., moderate to high in the meta-analysis for total cancer mortality (*I*^2^ 58%); thus, we have summarized the results across studies using random-effect model, which provides a wider confidence interval of the summary HR and more conservative claims of statistical significance compared to fixed-effect models. We could not explore the sources of heterogeneity between studies due to limited number of studies. Third, studies were conducted in US, Australia and United Kingdom, while limiting generalizability, particularly to low- and middle-income countries. Finally, our risk of bias assessment suggests that most of the studies are prone to bias, particularly confounding bias and selective reporting bias. For instance, most studies did not adjust for dietary factors (e.g., red and processed meat) that may play a role in cancer incidence and mortality. Residual confounding by smoking is also a concern in physical activity and cancer studies [26, 28]. Selective reporting of a particular outcome, analysis or subset of participants may lead to bias if based on direction, magnitude of the association or statistical significance of results [15]. All studies included in our review did not provide an analysis specified in a protocol or statistical analysis plan, before analyses were carried out, to be graded as low risk of bias. Future studies should embrace reproducible research practices (e.g., protocol registration) to reduce reporting bias.

## Conclusion

In conclusion, our systematic literature review suggests that muscle-strengthening activities might play a preventive role for incidence of kidney cancer. In addition, we found that muscle-strengthening activities might be a protective factor for total cancer mortality, and that performing both muscle-strengthening and aerobic activities may provide a larger reduction in total cancer mortality. The associations between muscle-strengthening and incidence of other types of cancer remain inconclusive due to the limited number of studies and moderate to high heterogeneity between results. Future prospective cohort studies with long-term follow-up, repeated-measures of muscle-strengthening activities over time and the intensity they are performed, and detailed covariates including aerobic MVPA should be conducted to provide a stronger evidence-base for synthesis. In addition, studies including more diverse populations with wide range of muscle-strengthening activities are needed to increase generalizability and to define dose-response relationship of muscle-strengthening activities with cancer incidence and mortality.

## Supplementary Information

**Additional file 1.**

## Data Availability

All data generated as part of this systematic review are included as additional files in this published article.

## Abbreviations

CIs: Confidence intervals
HR: Hazard ratio
MVPA: Moderate to vigorous physical activity
WHO: World Health Organization
