# Supplementary material for: Muscle-strengthening activities and cancer incidence and mortality: a systematic review and meta-analysis of observational studies
Source: Int J Behav Nutr Phys Act. 2021 May 29;18:69. doi: 10.1186/s12966-021-01142-7 (PMC8164763; doi:10.1186/s12966-021-01142-7)
Supplement: Supplementary file 1 — Additional file 1. [file 12966_2021_1142_MOESM1_ESM.docx]

**SUPPLEMENTARY MATERIALS**

**SEARCH STRATEGY:**

**Pubmed/Medline – 03/03/2020 – 1114 records**

(((((((((((((“resistance train*”) OR (“resistance exercise*”)) OR (“strength train*”)) OR (“strength exercise*”)) OR (“strengthening programs”)) OR (“weight bearing exercise”)) OR (“weight exercise*”)) OR (“weight train*”)) OR ("circuit training")) OR ("isometric exercise*")) OR ("strength endurance*")) OR ("weight bearing strengthening")) OR ("weight lifting")) AND (((((((((((sarcoma) OR (adenocarcinoma)) OR (malignan*)) OR (tumour*)) OR (tumor*)) OR (carcinom*)) OR (neoplas*)) OR (oncolog*)) OR (cancer*)) OR (Carcinoma)) OR (Neoplasms)) Filters: Humans

**Web of Science - 03/03/2020 – 1601 records**

(((((((((((((“resistance train*”) OR (“resistance exercise*”)) OR (“strength train*”)) OR (“strength exercise*”)) OR (“strengthening programs”)) OR (“weight bearing exercise”)) OR (“weight exercise*”)) OR (“weight train*”)) OR ("circuit training")) OR ("isometric exercise*")) OR ("strength endurance*")) OR ("weight bearing strengthening")) OR ("weight lifting")) AND (((((((((((sarcoma) OR (adenocarcinoma)) OR (malignan*)) OR (tumour*)) OR (tumor*)) OR (carcinom*)) OR (neoplas*)) OR (oncolog*)) OR (cancer*)) OR (Carcinoma)) OR (Neoplasms))

**Scopus - 03/03/2020 – 2549 records**

( TITLE-ABS-KEY ( sarcoma ) ) OR ( TITLE-ABS-KEY ( adenocarcinoma ) ) OR ( TITLE-ABS-KEY ( malignan* ) ) OR ( TITLE-ABS-KEY ( tumour* ) ) OR ( TITLE-ABS-KEY ( tumor* ) ) OR ( TITLE-ABS-KEY ( carcinom* ) ) OR ( TITLE-ABS-KEY ( neoplas* ) ) OR ( TITLE-ABS-KEY ( oncolog* ) ) OR ( TITLE-ABS-KEY ( cancer* ) ) OR ( TITLE-ABS-KEY ( carcinoma ) ) OR ( TITLE-ABS-KEY ( neoplasms ) ) AND ( ALL ( "resistance train*" ) ) OR ( TITLE-ABS-KEY ( "resistance exercise*" ) ) OR ( TITLE-ABS-KEY ( "strength train*" ) ) OR ( TITLE-ABS-KEY ( "strength exercise*" ) ) OR ( TITLE-ABS-KEY ( "strengthening programs" ) ) OR ( TITLE-ABS-KEY ( "weight bearing exercise" ) ) OR ( TITLE-ABS-KEY ( "weight exercise*" ) ) OR ( TITLE-ABS-KEY ( "weight train*" ) ) OR ( TITLE-ABS-KEY ( "circuit training" ) ) OR ( TITLE-ABS-KEY ( "strength endurance*" ) ) OR ( TITLE-ABS-KEY ( "weight bearing strengthening" ) ) OR ( TITLE-ABS-KEY ( "weight lifting" ) ) AND ( LIMIT-TO ( SRCTYPE , "j" ) ) AND ( LIMIT-TO ( DOCTYPE , "ar" ) ) AND ( LIMIT-TO ( EXACTKEYWORD , "Human" ) OR LIMIT-TO ( EXACTKEYWORD , "Humans" ) )

**Embase – 19/02/2020 – 2687 records**

('resistance training'/exp OR 'resistance training' OR 'resistance exercise'/exp OR 'resistance exercise' OR 'strength training'/exp OR 'strength training' OR 'strength exercise'/exp OR 'strength exercise' OR 'strengthening program' OR 'weight bearing exercise'/exp OR 'weight bearing exercise' OR 'weight exercise' OR 'weight training'/exp OR 'weight training' OR 'circuit training'/exp OR 'circuit training' OR 'isometric exercise'/exp OR 'isometric exercise' OR 'strength endurance' OR 'weight bearing strengthening' OR (('weight' OR 'weight'/exp OR weight) AND bearing AND strengthening) OR 'weight lifting'/exp OR 'weight lifting' OR 'muscle exercise'/exp OR 'muscle exercise') AND ('sarcoma'/exp OR 'sarcoma' OR 'adenocarcinoma'/exp OR 'adenocarcinoma' OR 'malignant neoplasm'/exp OR 'malignant neoplasm' OR 'tumour' OR 'tumour'/exp OR tumour OR 'tumor' OR 'tumor'/exp OR tumor OR 'carcinoma'/exp OR 'carcinoma' OR 'neoplasm'/exp OR 'neoplasm' OR 'neoplasia'/exp OR 'neoplasia' OR 'oncology'/exp OR 'oncology' OR 'cancer' OR 'cancer'/exp OR cancer OR 'neoplasms' OR 'neoplasms'/exp OR neoplasms) AND [embase]/lim AND 'human'/de

**Table S1**: Main characteristics of the twelve studies included in the systematic review

| **Author/**  **Year/**  **Country/**  **Observation** | **Participants characteristics:** | **Study Design** | **Measurement of exposure:** | **Outcome** | **Exclusion criteria and**  **Multivariable models** | **Main results** |
| --- | --- | --- | --- | --- | --- | --- |
| Dankel, 2017, National Health and Nutrition Examination Survey 1999-2002,  USA^25^  Obs: Included in the meta-analysis displayed in the Figure 3 | **sample size:**  2773  **Sex:**  Men (49,6%) and Women (50,4%)  **Age at baseline**  Over 50 years | Cohort  **Average years:**  9.7 (sd 0.04) years;  **Pearson-years:**  26,898 py | **Type of measure:**  Self-reported:  **Type of strength training:**  Weight lifting/strength training;  **Categories:**  Knee extensor strength (dichotomous; 75th percentile vs. below), lowest quartile with those in the upper three quartiles.  and engagement in muscle  strengthening activities (dichotomous; ≥8 sessions per week vs. <8 sessions per week)  **Joint analysis:**  No | **Type**:  Mortality  **Cancer site:**  All cancer | **Excluded unhealthy participants:**  Die during the first 12-months of the follow-up period  **Model 1:**  Age, gender, race/ethnicity, and aerobic physical activity  **Model 2:**  Model 1 plus total cholesterol, mean arterial pressure, body mass index, C-reactive protein, self-reported smoking status, use of ambulatory device, statin medication, arthritis, congestive heart failure, coronary artery disease, cancer, diabetes, and stroke | **Cancer mortality:**  162 deaths  **HR and 95% CI (Model 2)**  Sufficient engagement in muscle strengthening activities vs. not  0.92(0.45, 1.86) |
| Loprinzi, 2017,^20^  National Health and Nutrition Examination Survey 2003-2006,  USA  Obs: Included in the meta-analysis displayed in the Figure 3 | **sample size:** 1411  **Sex:** Men (39,1%) and Women (60,9%)  **Age at baseline**  58.9 years | Cohort  **Average years:** 6.75 years  **Pearson-years:** 9,271 | **Type of measure:** Self-reported:  **Type of strength training: “**Activities designed to strengthen your muscles (such as lifting weights, push-ups, or sit-ups)  **Categories:** Those meeting muscle strengthening guidelines (>2/week were considered to meet muscle strengthening guidelines) vs. not (<2/week were considered to meet muscle strengthening guidelines)”  **Joint analysis:** no | **Type**:  Mortality  **Cancer site:**  All cancers | **Excluded unhealthy participants:**  excluded if they died within the first 12-months of the follow-up period or had any of the following conditions at baseline: coronary artery disease, congestive heart failure or stroke.  **Model 1**:  Adjusted for aerobic-based physical activity, age, gender, race-ethnicity, smoking, C-reactive protein, cholesterol medication use, hypertension, diabetes and body mass index | **Cancer mortality:**  60 deaths  **HR and 95% CI (Model 1)**  Meeting muscle strengthening physical activity (vs. not) - 0.27 (0.06–1.20) |
| Kamada,  2017,^8^  Women’s Health Study,  USA  Obs: Included in the meta-analysis displayed in the Figure 3 and 4 | **sample size:** 28879  **Sex:** Women only  **Age at baseline:** 62.2 years | Cohort  **Average years:** 12 (sd 3.2) years  **Pearson-years:** 346,843 | **Type of measure:** Self-reported:  **Type of strength training:** Weight lifting/strength training;  **Categories:** 0, 1-59, and ≥60 min/weeks  **Joint analysis:** Yes | **Type**:  Mortality  **Cancer site:**  All cancers | **Excluded unhealthy participants:**  CVD (myocardial infarction, stroke, percutaneous transluminal coronary angioplasty, or coronary artery bypass grafting), cancer, or diabetes mellitus.  **Model 1**:  Age and trial randomization  **Model 2**:  Additionally adjusted for postmenopausal status, hormone use, smoking status, parental history of myocardial infarction or cancer, alcohol intake, energy intake, saturated fat intake, fiber intake, fruit and vegetable intake, and time per week spent in aerobic moderate-to-vigorous physical activity.  **Model 3**:  Additionally adjusted for body mass index and incidence of hypertension, high cholesterol, CVDs, diabetes mellitus, and cancer before and during follow-up | **Cancer mortality:**  748 deaths  **HR and 95% CI (Model 2)**  0 min/wk:  1.00 ( reference)  1-59 min/wk:  0.88 (0.73,1.05)  ≥60 min/wk:  0.99 (0.73, 1.33)  Aerobic and Strength Training:  Aerobic MVPA <150 Min/Wk and No ST - Reference  Aerobic MVPA ≥150 Min/Wk and No ST - 0.97 (0.79–1.19)  Aerobic MVPA <150 Min/Wk and Any ST - 0.91 (0.70–1.18)  Aerobic MVPA ≥150 Min/Wk and Any ST - 0.93 (0.74–1.17) |
| Kraschnewski, 2016,^18^  National Health Interview Survey,  USA  Obs. Not included in the meta-analysis due to fewer participants compared to Siahpush et al.) | **sample size:** 30162  (25,663 adjusted model)  **Sex:** Men (42,4%) and Women (57,6%)  **Age at baseline:**74.2 years | Cohort  **Average years:** 15 years  **Pearson-years:** Not reported | **Type of measure:** Self-reported:  **Type of strength training: “**Frequency of leisure-time physical activities specifically designed to strengthen your muscles (such as lifting weight or doing calisthenics)”  **Categories:**  >2 times/week and ≥2 times/week  **Joint analysis:** no | **Type**:  Mortality  **Cancer site:**  All cancers | **Excluded unhealthy participants:**  No exclusions  **Model 1**:  Demographics (age, gender, race, ethnicity(hispanic, non-hispanic), educational status, marital status)  **Model 2**:  Additionally adjusted for health behaviors(Body mass index, Physical activity, Alcohol use, Smoking status)  **Model 3**:   Additionally adjusted for comorbid conditions(Diabetes, Hypertension, Coronary heart disease, Non-skin cancer) | **Cancer mortality:**  2192 deaths  **OR and 95% CI (Model 3)**  ST 2+ t/wk vs. ST <2 t/wk - 0.84(0.68, 1.03) |
| Zhao,  2020,^21^  National Health Interview Survey,  USA  Obs. Included only in the meta-analysis displayed in the Figure 4. | **sample size:**  479856  **Sex:**  Men (44,9%) and  Women (55,1%)  **Age at baseline**  Over 18 years | Cohort  **Average years:**  8.75 years  **Pearson-years:**  Not reported | **Type of measure:** Self-reported:  **Type of strength training:** Physical activities specifically designed to strengthen your muscles (such as lifting weights or doing calisthenic)  **Categories:** recommended activity (≥150 minutes of light to moderate intensity activity each week, or ≥75 minutes of vigorous intensity activity, or greater than or equal to an equivalent combination) and insufficient activity (<150 minutes of light to moderate intensity activity each week and <75 minutes of vigorous intensity activity, and less than an equivalent combination). Muscle strengthening activity was also categorized into two groups: recommended (≥2 times/week) and insufficient (<2 times/week).  **Joint analysis:** Yes | **Type**:  Mortality  **Cancer site:**  All cancers | **Excluded unhealthy participants:**  Were excluded because of pregnancy, missing data on  aerobic physical activity or muscle strengthening activity, and missing data on potential covariates (personal variables, lifestyle factors, or chronic health conditions)  **Model 1:** Adjusted for sex, age, and race/ethnicity.  **Model 2:** Model 1+education, marital status, body mass index, smoking status, and alcohol intake.  **Model 3:** Model 2+chronic conditions. | **Cancer mortality:**  14375 deaths  **HR and 95% CI (Model 3)**  Insufficient aerobic or muscle strengthening - 1.00  Muscle strengthening only - 0.85(0.77, 0.95)  Aerobic only - 0.76(0.73, 0.80)  Aerobic and muscle strengthening - 0.60(0.56, 0.65) |
| Siahpush, 2019,^24^  National Health Interview Survey,  USA  Obs: Included in the meta-analysis displayed in the Figure 3 | **sample size:** 310628  **Sex:** Men (48,6%) and  Women (51,4%)  **Age at baseline:** Over 18 years | Cohort  **Average years:** 7.9 years  **Pearson-years:** Not reported | **Type of measure:** Self-reported:  **Type of strength training:** Physical activities specifically designed to strengthen your muscles such as lifting weights or doing calisthenics;  **Categories:** Strengthening exercises≥ 2 times per week or less. In order to assess the presence of a dose–response relationship between MSA and cancer mortality, we recategorized MSA performance to zero, one, two, three, four, and five times or more often a week.  **Joint analysis:** No | **Type**:  Mortality  **Cancer site:**  All cancers | **Excluded unhealthy participants:**  None  **Model 1:** adjust for the effect of minutes of moderate physical activity, minutes of vigorous physical activity, smoking status, BMI, previous cancer diagnosis, chronic condition, self-rated health, sex, age, marital status, race/ethnicity, nativity, poverty status, and education | **Cancer mortality:**  7275 deaths  **HR and 95% CI (Model 1)**  ST 2+ t/wk vs. ST <2 t/wk -  0.81 (0.73, 0.89) |
| Stamatakis, 2017,^11^  The Health Survey for England and the Scottish Health  Survey  Obs: Included in the meta-analyses displayed in the Figures 3 and 4 | **sample size:** 77195  **Sex:** Men (43,6%) and  Women (54,4%)  **Age at baseline:** Over 30 years | Cohort  **Average years:** 9.2 (sd 4.5) years  **Pearson-years:** 736,463 | **Type of measure:** Self-reported:  **Type of strength training:** Own-body-weight and gym-based strength-promoting;  **Categories:** None, any, low (<sex-specific median values), high (>sex-specific median values) and adherence to the SPE guideline (was defined as reporting participation in at least 2 sessions per  week, on average).  **Joint analysis:** Yes | **Type**:  Mortality  **Cancer site:**  All cancers | **Excluded unhealthy participants:**  Excluded participants who died during the first 24 months of follow-up. We excluded persons with prevalent cancer at baseline from the cancer mortality analyses; persons with prevalent CVD (angina, stroke, or ischemic heart disease) from the CVD mortality analyses; and both persons with prevalent CVD and persons with prevalent cancer from the all-cause mortality analyses.  **Model 1:**  Adjusted for age and sex.  **Model 2:**  Also adjusted for long-standing illness, frequency of alcohol consumption, psychological distress, body mass index,  smoking status, educational level, and weekly volume of physical activity, excluding the volume of strength-promoting activity that was the main exposure in the corresponding model. | **Cancer mortality:**  2089 deaths  **HR and 95% CI (Model 2)**  Own-body-weight activity  None - 1.00  Any - 0.69(0.56, 0.86)  Weekly volumed  None - 1.00  Low - 0.66(0.47, 0.92)  High - 0.72(0.54, 0.96)  Gym-based activity  None - 1.00  Any - 0.61(0.45, 0.84)  Weekly volumed  None - 1.00  Low - 0.66(0.44, 0.98)  High - 0.56(0.34, 0.91)  All strength exercise  None - 1.00  Any - 0.69(0.57, 0.84)  Weekly volumed  None - 1.00  Low - 0.72(0.58, 0.93)  High - 0.67(0.52, 0.88)  Adherence to strength exercise guideline  Did not meet the guideline - 1.00  Met the guideline - 0.80(0.70, 0.91)  Strength and aerobic guidelines  Neither - 1.00  Only strength - 0.66(0.48, 0.92)  Only aerobic - approximately 1.00  Both - 0.70(0.50, 0.98) |
|  |  |  |  |  |  |  |
|  |  |  |  |  |  |  |
| Patel, 2020,^19^  Cancer Prevention Study-II Nutrition  Cohort,  USA  Obs: Included in the meta-analysis displayed in the figure 3 | **sample size:** 72462  **Sex:** Men and Women  **Age at baseline:** mean 70.2; standard deviation ≥6.0 | Cohort  **Average years:** 13 years  **Pearson-years:** 858,203 | **Type of measure:** Self-reported:  **Type of strength training:** weight training/resistance exercises  **Categories:** None, >0 to <1 hour/week, 1 to <2 hours/week, ⩾2 hours/week | **Type**:  Mortality  **Cancer site:**  All cancers | **Excluded unhealthy participants:**  personal history of cancer, cardiovascular disease, stroke, emphysema, or lung disease; were missing body mass index (BMI) (weight in kg/height in m^2^), MVPA, MSA, or smoking status; had a date of death that preceded the date of the survey return; or self-reported poor health  **Model 1** is adjusted for sex and age (single year).  **Model 2** is adjusted for sex and age, and additionally adjusted for BMI, survey type (long/short), education, self-reported overall health, smoking duration and intensity, alcohol use, marital status, work status, TV sitting time, aspirin use, and comorbidity score (reported personal history of high blood pressure, type 2 diabetes, and high cholesterol).  **Model 3** includes all covariates from Model 2 and additionally adjusts for aerobic moderate-to-vigorous physical activity (<7.5, 7.5 - <15, ≥15 h/wk) | **Cancer mortality:**  5038 deaths  **HR and 95% CI (Model 3)**  ≥2 h vs none -  1.02(0.89, 1.17) |
| Rezende, 2020,^13^  Health Professionals Follow-up Study,  USA  Obs: Included in the meta-analyses displayed in the Figure 2 and 4 | **sample size:** 33787  **Sex:** Men only  **Age at baseline:** 40 to 75 years | Cohort  **Average years:** 15 years  **Pearson-years:** 521,221 | **Type of measure:** Self-reported:  **Type of strength training:** Weight lifting or nautilus or weight machine;  **Categories:** None, <1 hour/week and 1+ hour/week and Per 1-h/week increase [continuous (per hour a week)]  **Joint analysis:** Yes | **Type**:  Incidence  **Cancer site:**  All cancers, colon (ICD-9 153), advanced prostate cancer (ICD-9 185; i.e., advanced prostate cancer was defined as stage T3b, T4, N1, M1, or death from prostate cancer), lung (ICD-9 162), bladder (ICD-9 188), lymphoma (ICD-9 200, 202, 204), pancreas (ICD-9 157), kidney (ICD-9 189), leukemia (ICD-9 205-207), multiple myeloma (ICD-9 203), and esophageal (ICD-9 150). | **Excluded unhealthy participants:**  Excluded men having prior  cancer diagnosis.  **Model 1:**  Age-adjusted[Cox regression models using age (month) as time scale with stratification by calendar time (year)], additionally adjusted for race (white or non-white), height (continuous), family history of cancer (yes or no), physical exam in past two  years (yes or no), history of colonoscopy or sigmoidoscopy (yes or no), smoking in pack years (never smoker, 1–4.9, 5–19.9, 20–39.9 or ≥40), regular aspirin use  (yes or no), multivitamin use (yes or no), alcohol consumption (0, 0.1–4.9, 5.0–14.9, 15.0–29.9, or ≥30 g/d), red and processed meat intake (quintiles), Alternate  Healthy Eating Index (quintiles) and prostate-specific antigen test in past 2 years (yes or no).  **Model 2:**  Additionally adjusted for total physical activity except for resistance training (quintiles).  **Model 3:**   Additionally adjusted for total energy intake (quintiles) and body mass index (quintiles) | **Cancer cases:**  5158 cases  **HR and 95% CI (Model 2)**  **Total cancer**  5158 cases  None - 1.00  <1 hour/week - 0.97 (0.90, 1.05)   1+ hour/week - 0.98 (0.89, 1.09)  Per 1-h/week increase - 1.01 (0.97, 1.05)  **Colon cancer**  700 cases  None - 1.00  <1 hour/week - 0.94 (0.76, 1.15)   1+ hour/week -1.28 (0.98, 1.67)  Per 1-h/week increase - 1.11 (1.02, 1.22)  **Advanced prostate cancer**  657 cases  None - 1.00  <1 hour/week - 0.95 (0.76, 1.19)   1+ hour/week - 0.88 (0.66, 1.19)  Per 1-h/week increase - 0.95 (0.84, 1.07)  **Lung cancer**  595 cases  None - 1.00  <1 hour/week - 0.87 (0.69, 1.09)   1+ hour/week - 0.90 (0.64, 1.28)  Per 1-h/week increase - 0.93 (0.79, 1.09)  **Bladder cancer**  505 cases  None - 1.00  <1 hour/week - 0.94 (0.75, 1.17)   1+ hour/week - 0.61 (0.42, 0.89)  Per 1-h/week increase - 0.80 (0.66, 0.96)  **Lymphoma**  484 cases  None - 1.00  <1 hour/week - 1.02 (0.81, 1.29)   1+ hour/week - 1.08 (0.79, 1.49)  Per 1-h/week increase - 1.06 (0.95, 1.19)  **Pancreatic cancer**  233 cases  None - 1.00  <1 hour/week - 1.11 (0.79, 1.55)   1+ hour/week - 1.18 (0.74, 1.89)  Per 1-h/week increase - 1.01 (0.83, 1.23)  **Kidney cancer**  212 cases  None - 1.00  <1 hour/week - 0.88 (0.62, 1.25)   1+ hour/week - 0.57 (0.31, 1.03)  Per 1-h/week increase - 0.77 (0.58, 1.03)  **Leukemia**  188 cases  None - 1.00  <1 hour/week - 0.80 (0.55, 1.18)   1+ hour/week - 0.99 (0.58, 1.68)  Per 1-h/week increase - 1.09 (0.90, 1.32)  **Multiple myeloma**  112 cases  None - 1.00  <1 hour/week - 0.98 (0.60, 1.59)   1+ hour/week - 0.91 (0.45, 1.85)  Per 1-h/week increase - 0.85 (0.58, 1.23)  **Oesophageal cancer**  103 cases  None - 1.00  <1 hour/week - 1.28 (0.78, 2.10)   1+ hour/week - 0.72 (0.30, 1.74)  Per 1-h/week increase - 0.92 (0.65, 1.30)  Joint analysis  Colon  None resistance training  Low - 1.00  High - 0.88(0.73, 1.06)  Any resistance training  Low - 084(0.57, 1.25)  High - 0.95(0.77, 1.19)  Bladder  None resistance training  Low - 1.00  High - 1.19(0.95, 1.50)  Any resistance training  Low - 0.75(0.46, 1.21)  High - 1.03(0.8, 1.35)  Kidney  None resistance training  Low - 1.00  High - 0.87(0.62, 1.21)  Any resistance training  Low - 1.02(0.53, 1.87)  High - 0.65(0.43, 0.97) |
| Mazzilli, 2019,^12^  National  Institutes of Health (NIH)-American Association of Retired  Persons (AARP) Diet and Health Study,  USA  Obs: Included in the meta-analyses displayed in the Figure 2 and 4 | **sample size:** 215,122  **Sex:** Men (56,3%) and  Women (43,7%)  **Age at baseline:** 50 to 71 years | Cohort  **Average years:** 10 years  **Pearson-years:** Not reported | **Type of measure:** Self-reported:  **Type of strength training:** Weight lifting/strength training;  **Categories:** None (0 min), Low (5 min to 1.5 h per wk), high(2–10+ h per wk) Secondary analyze colon cancer divided in none and any  **Joint analysis:** Yes | **Type**:  Incidence  **Cancer site:**  colon (C180-C189, C260), kidney (C649 and C659), bladder (C670-C679), breast (C500-C509), lung (C340-C349),non-Hodgkin lymphoma (C024, C098, C099, C111, C142, C379, C422, C770-C779), pancreatic (C250-C259), prostate (C619), rectum (C199, C209), and malignant melanoma (C440-C449). | **Excluded unhealthy participants:**  Participants who moved  out of the cancer registry catchment area before follow-up  ,proxy respondents, those who  self-reported cancers before completion of the follow-up questionnaire including; ovarian ,endometrial,prostate, colorectal, lung, breast , pancreas, non-Hodgkin lymphoma, melanoma and participants with a cancer diagnosis before the follow-up questionnaire.  **Model 1:**  Adjusted for age, sex, BMI, smoking status, race, education, alcohol intake.  **Model 2:** Additionally adjusted  vigorous leisure time physical activity not including weight lifting (MVPA).  For Breast: Additionally adjusted for oral birth control use, age of menarche, age of menopause, postmenopausal hormone use, and parity. | **Cases of Cancer:**  23,346  **HR and 95% CI (Model 2)**  **Colon**  1715 cases  None - 1.00  Low - 0.75(0.66, 0.87)  High - 0.78(0.61, 0.98)  **Kidney**  851 cases  None - 1.00  Low - 0.94(0.78, 1,12)  High - 0.80(0.59, 1.11)  **Bladder**  1836 cases  None - 1.00  Low - 0.97(0.86, 1.10)  High - 0.98(0.81, 1,19)  **Breast**  3288 cases  None - 1.00  Low - 1.02(0.93, 1.11)  High - 0.99(0.83, 1.17)  **Lung**  3480 cases  None - 1.00  Low - 0.91(0.82, 1.00)  High - 0.90(0.81, 1.12)  **Non-Hodgkin’s lymphoma**  1187 cases  None - 1.00  Low - 0.90(0.78, 1.05)  High - 0.96(0.75, 1.23)  **Pancreas**  795 cases  None - 1.00  Low - 1.15(0.96, 1.37)  High - 0.98(0.71, 1.34)  **Prostate**  7213 cases  None - 1.00  Low - 1.03(0.97, 1.09)  High - 1.05(0.96, 1.15)  **Rectum**  527 cases  None - 1.00  Low - 0.68(0.52, 0.88)  High - 1.01(0.69, 1.48)  **Melanoma**  2454 cases  None - 1.00  Low - 1.18(1.07, 1.30)  High - 1.03(0.88, 1.20)    **Joint association**  Colon cancer in relation to weight lifting, comparing any vs no weight lifting  All participants - 0.95 (0.90, 1.00)  Male - 0.91 (0.84, 0.98)  Female - 1.00 (0.93,1.08)  Colon cancer risk by any vs no  weight lifting and low vs high leisure time moderate to vigorous physical activity  All participants  No Weight Lifting  Low Activity - 1.00  High Activity - 0.93 (0.83, 1.03)  No Weight Lifting  Low Activity - 0.77 (0.57, 1.03)  High Activity - 0.69 (0.60, 0.80)  Men  No Weight Lifting  Low Activity - 1.00  High Activity - 0.87 (0.76, 1.00)  No Weight Lifting  Low Activity - 0.69 (0.47, 1.02)  High Activity - 0.61 (0.50, 0.73)  Women  No Weight Lifting  Low Activity - 1.00  High Activity - 1.01 (0.85, 1.19)  No Weight Lifting  Low Activity - 0.88 (0.55, 1.41)  High Activity - 0.83 (0.66, 1.04) |
| Boyle, 2012,^22^  case–control study of  colorectal cancer in Western Australia,  Australia  Obs: Included in the meta-analysis displayed in the Figure 2 | **sample size:** 1866  **Sex:** Men and Women  **Age at baseline:** 40 to 79 years | Case-control  **Average years:**  Not applicable  **Pearson-years:**  Not applicable | **Type of measure:** Self-reported:  **Type of strength training:** Definitely (‘‘resistance training,’’ ‘‘weights,’’ ‘‘weight training,’’ ‘‘strength training,’’ ‘‘circuit training’’ and ‘‘weight lifting’’), possibly (‘‘gym,’’ ‘‘gym activities,’’ ‘‘health club,’’  and ‘‘home gym’’), or never;  **Categories:** Definitely, possibly,  or never performing resistance training in each age period and over the lifetime.  **Joint analysis:** no | **Type**:  Incidence  **Cancer site:**  Colon and rectal. | **Excluded unhealthy participants:**  Omitted participants with missing information on one or more covariates.  **Model 1:**  Adjusted for age group and sex only.  **Model 2:**  Adjusted additionally for resistance training in other age periods, lifetime moderate and vigorous non-resistance training recreational physical activity, lifetime occupational activity, and energy intake | **Cases of Cancer:**  870 (and 996 controls)  **OR and 95% CI (Model 2)**  **Colon cancer**  552 cases  19 to 34 years  Never 1.00  Definite 0.74 (0.33–1.66)  Possible 0.74 (0.30–1.86)  Definite or possible combined 0.74 (0.40–1.35)  35 to 50 years  Never 1.00  Definite 0.61 (0.29–1.31)  Possible 0.74 (0.39–1.38)  Definite or possible combined 0.69 (0.42–1.12)  51+ years  Never 1.00  Definite 0.80 (0.43–1.50)  Possible 0.76 (0.41–1.38)  Definite or possible combined 0.78 (0.51–1.22)  Adult lifetime  Never 1.00  Definite 0.70 (0.45–1.11)  Possible 0.67 (0.43–1.04)  Definite or possible combined 0.68 (0.49–0.95)  **Rectal cancer**  318 cases  19 to 34 years  Never 1.00  Definite 1.29 (0.60–2.82)  Possible 1.36 (0.53–3.48)  Definite or possible combined 1.36 (0.75–2.48)  35 to 50 years  Never 1.00  Definite 1.14 (0.54–2.38)  Possible 0.64 (0.29–1.41)  Definite or possible combined 0.86 (0.50–1.48)  51+ years  Never 1.00  Definite 0.58 (0.25–1.33)  Possible 0.67 (0.31–1.46)  Definite or possible combined 0.66 (0.37–1.16)  Adult lifetime  Never 1.00  Definite 1.16 (0.71–1.87)  Possible 0.78 (0.46–1.33)  Definite or possible combined 0.96 (0.66–1.39) |
| Keum, 2016,^23^  Health Professionals Follow-up Study,  USA  Obs: Not included in the meta-analyses. | **sample size:** 43479  **Sex:** Men only  **Age at baseline:** 40 to 75 years | Cohort  **Average years:**  Not reported  **Pearson-years:** 686,924 | **Type of measure:** Self-reported:  **Type of strength training:** Weight lifting;  **Categories:** Reference (1.4 median of PA, MET-h/wk), Q1(8.9 median of PA, MET-h/wk), Q2(22.4median of PA, MET-h/wk), Q3(47.5 median of PA, MET-h/wk) each one subdivided into weight lifting (yes or no).  **Joint analysis:** Yes | **Type**:  Incidence  **Cancer site:**  Digestive tract (mouth, throat, esophagus, stomach, small intestine, and colorectum), Digestive accessory organs (pancreas, gallbladder, and liver) and Digestive system(mouth, throat, esophagus, stomach, small intestine, colorectum, pancreas, gallbladder, and liver) | **Excluded unhealthy participants:**  Excluded men having a prior cancer diagnosis, body mass index (BMI), or dietary information. To  minimize potential bias from reverse causation, we also excluded men reporting difficulty with walking or stair climbing at any point during the follow-up.  **Model 1:**   Multivariable analyses were stratified by age (continuous) and questionnaire cycle; adjusted for Caucasian (yes vs. no), smoking (never, past, current, unknown status), family history of cancer(yes vs. no), history of physical examination for screening purpose (yes vs. no), current aspirin use (yes vs. no), current multivitamin use (yes vs. no), and intakes of total calories (quintiles), alcohol (0, 0.1-4.9, 5.0-29.9, 30+ g/day), red and processed meat (quintiles), whole grains (quintiles), fruits (quintiles), and vegetables (quintiles), and history of upper endoscopy (yes vs. no).  **Model 2:**  Additionally adjusted for BMI (<23, 23-24.9, 25-29.9, 30+ kg/m2).  **Model 3:**  Additionally adjusted for history of DM (yes vs. no). | **Cancer cases:**  1,370 (1,070 digestive tract cancers, 300 digestive accessory organ cancers)  **HR and 95% CI (Model 1)**  Joint Associations of Amount of Aerobic Exercise/Weight Lifting and Participation in Weight Lifting with Cancers  **Cancers of the Digestive Tract**  Participation in Weight Lifting - No(median, MET-hours/week)  717 cases  Reference(1.4) - 1.00  Q1(8.9) - 0.61(0.47,0.80)  Q2(22.4) - 0.58(0.44,0.77)  Q3(47.5) - 0.53(0.39,0.73)  Participation in Weight Lifting - Yes(median, MET-hours/week)  Reference(1.4) - 1.00  Q1(8.9) - 0.75(0.53,1.05)  Q2(22.4) - 0.58(0.42,0.80)  Q3(47.5) - 0.61(0.45,0.82)  **Cancers of the Digestive Accessory Organs**  Participation in Weight Lifting - No(median, MET-hours/week)  217 cases  Reference(1.4) - 1.00  Q1(8.9) - 0.73(0.44,1.23)  Q2(22.4) - 0.63(0.36,1.10)  Q3(47.5) - 0.62(0.34,1.14)  Participation in Weight Lifting - Yes(median, MET-hours/week)  Reference(1.4) - 1.00  Q1(8.9) - 0.89(0.47,1.68)  Q2(22.4) - 0.74(0.41,1.34)  Q3(47.5) - 0.65(0.36,1.15)  Joint Associations of Amount of Aerobic Exercise/Weight Lifting and Participation in Weight Lifting with **Digestive System Cancers**   Participation in Weight Lifting - No(median, MET-hours/week)  934 cases  Reference(1.4) - 1.00  Q1(8.9) - 0.64(0.50, 0.80)  Q2(22.4) - 0.59(0.46, 0.76)  Q3(47.5) - 0.54(0.41,0.72)  Participation in Weight Lifting - Yes(median, MET-hours/week)  Reference(1.4) - 1.00  Q1(8.9) - 0.77(0.57,1.04)  Q2(22.4) - 0.61(0.46, 0.81)  Q3(47.5) - 0.61(0.47,0.80) |


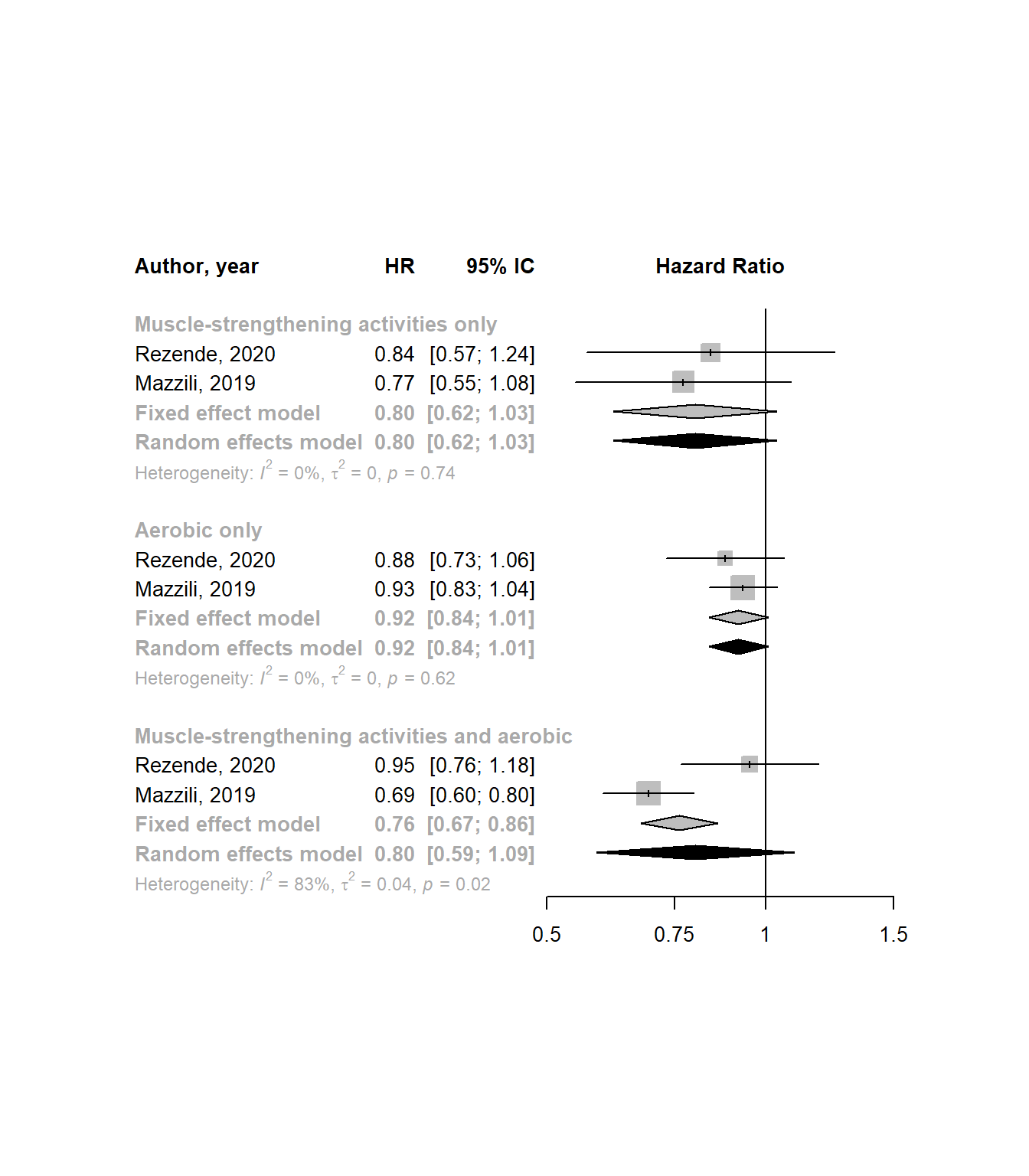


**Figure S1**: Meta-analysis for the joint association of muscle-strengthening activities and aerobic activities with colon cancer incidence.


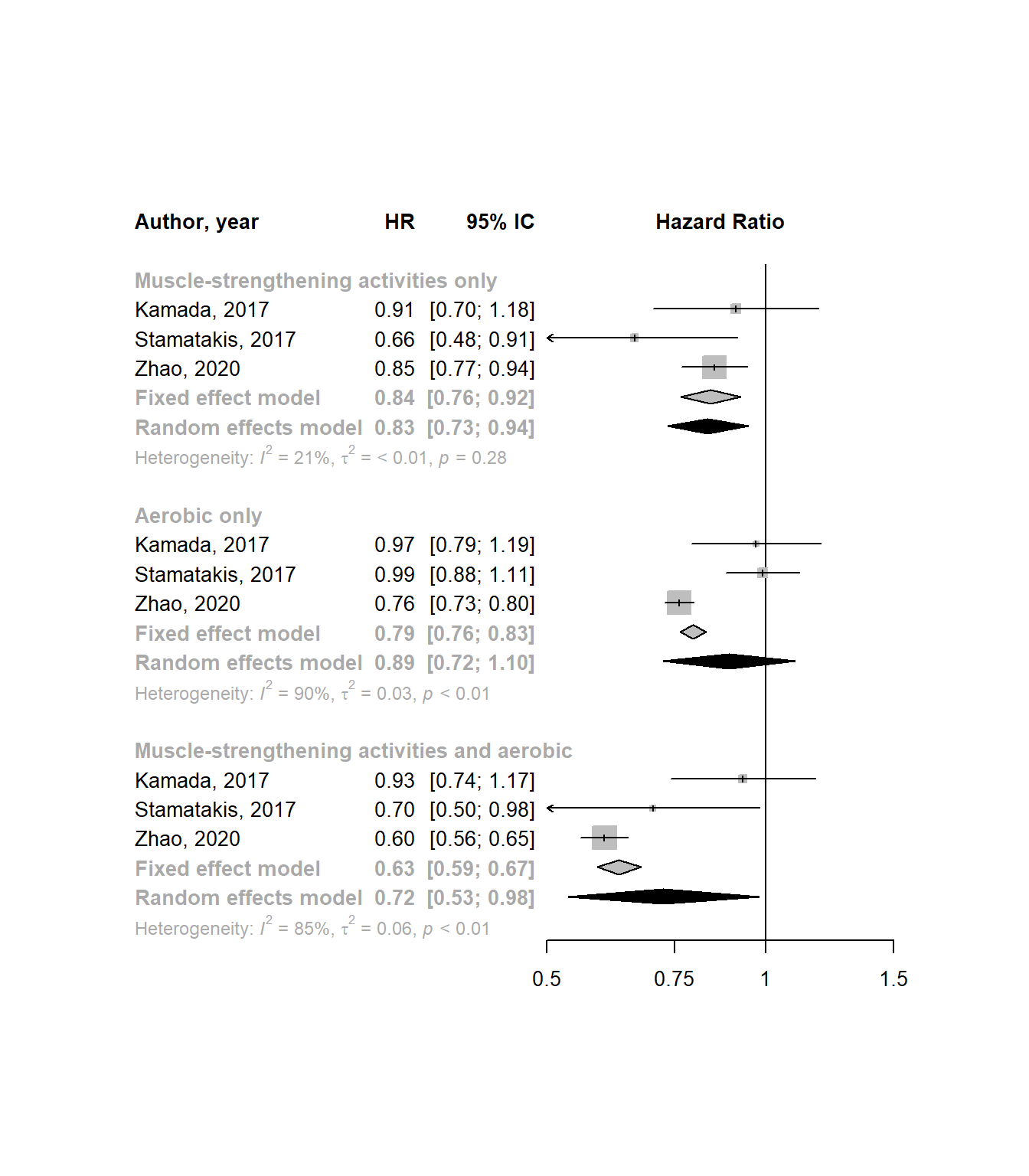


**Figure S1**: Meta-analysis for the joint association of muscle-strengthening activities and aerobic activities with cancer mortality.
